# Supplementary material for: Collagen promotes anti-PD-1/PD-L1 resistance in cancer through LAIR1-dependent CD8+ T cell exhaustion
Source: Nat Commun. 2020 Sep 9;11:4520. doi: 10.1038/s41467-020-18298-8 (PMC7481212; doi:10.1038/s41467-020-18298-8)
Supplement: Supplementary file 1 — Supplementary Information [file 41467_2020_18298_MOESM1_ESM.pdf]

Collagen promotes anti-PD-1/PD-L1 resistance in cancer through LAIR1-dependent $CD8^+$ T cell exhaustion

Peng et al.

Supplementary Figure 1. PD-(L)1 blockade is associated with increased tumor-associated collagen deposition

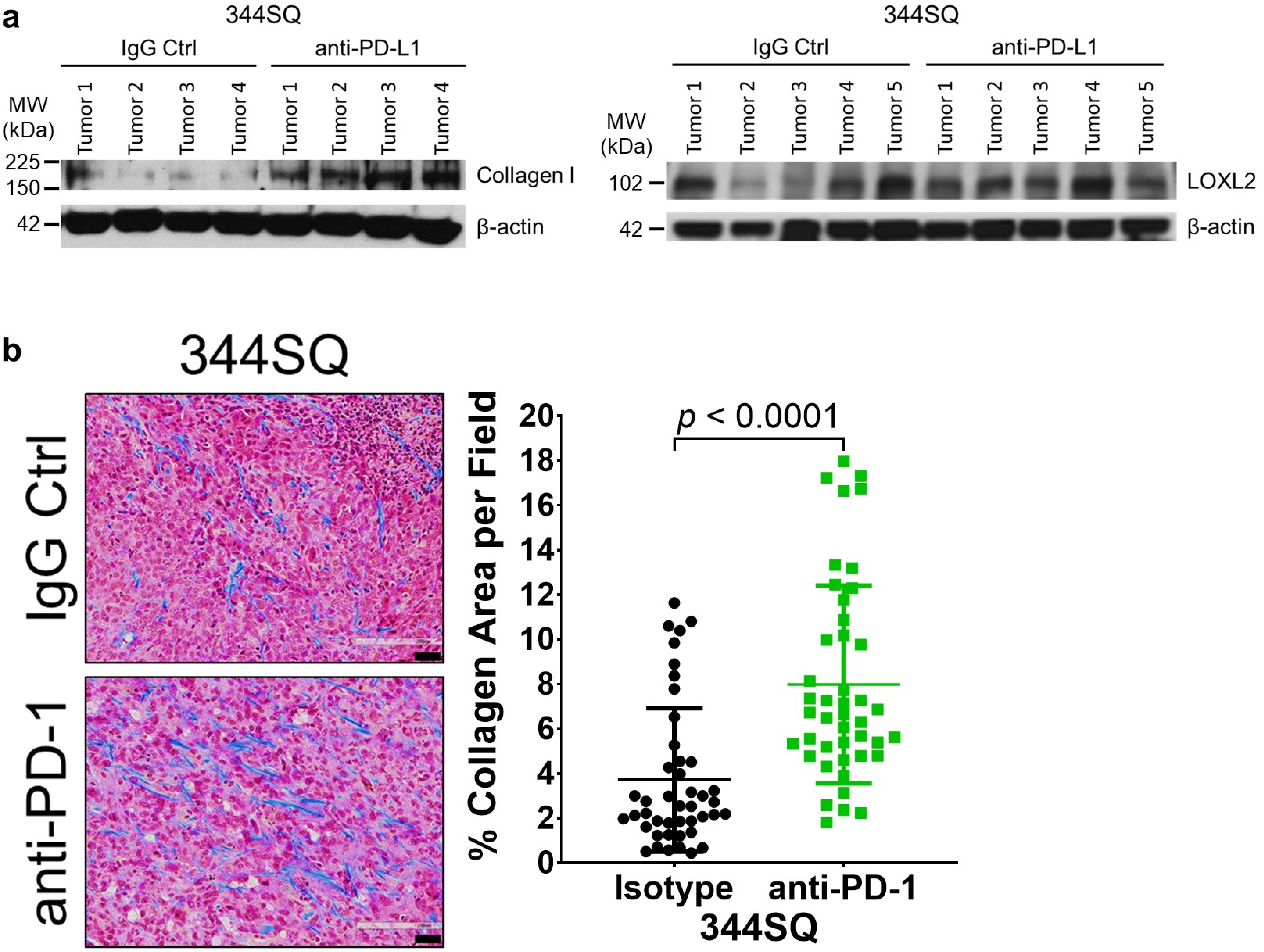

Supplementary Figure Legends

Supplementary Figure 1. PD-(L)1 blockade is associated with increased tumor-associated collagen deposition

(a) Western blots of collagen I, LOXL2, and β-actin loading control from representative 344SQ tumors treated weekly with anti-PD-L1 (200 µg/mouse) or IgG isotype control (200 µg/mouse). Western blots performed once for each experiment due to limited protein quantities.

(b) Representative trichrome stains including quantification of percent collagen area per field of 344SQ tumors treated weekly with IgG isotype control or anti-PD-1 (200 µg/mouse) after 1 week of implantation. Tumors were analyzed at endpoint of experiment; n = 5 tumors per treatment group and 44 total microscopy fields across all tumor samples were analyzed. Scale bars, 50 µm. Data presented as mean +/- SD. Statistics calculated using two-tailed student's t-test.

Supplementary Figure 2. LOXL2 knockdown or inhibition reduces intratumoral collagen crosslinking and deposition with no effect on tumor size

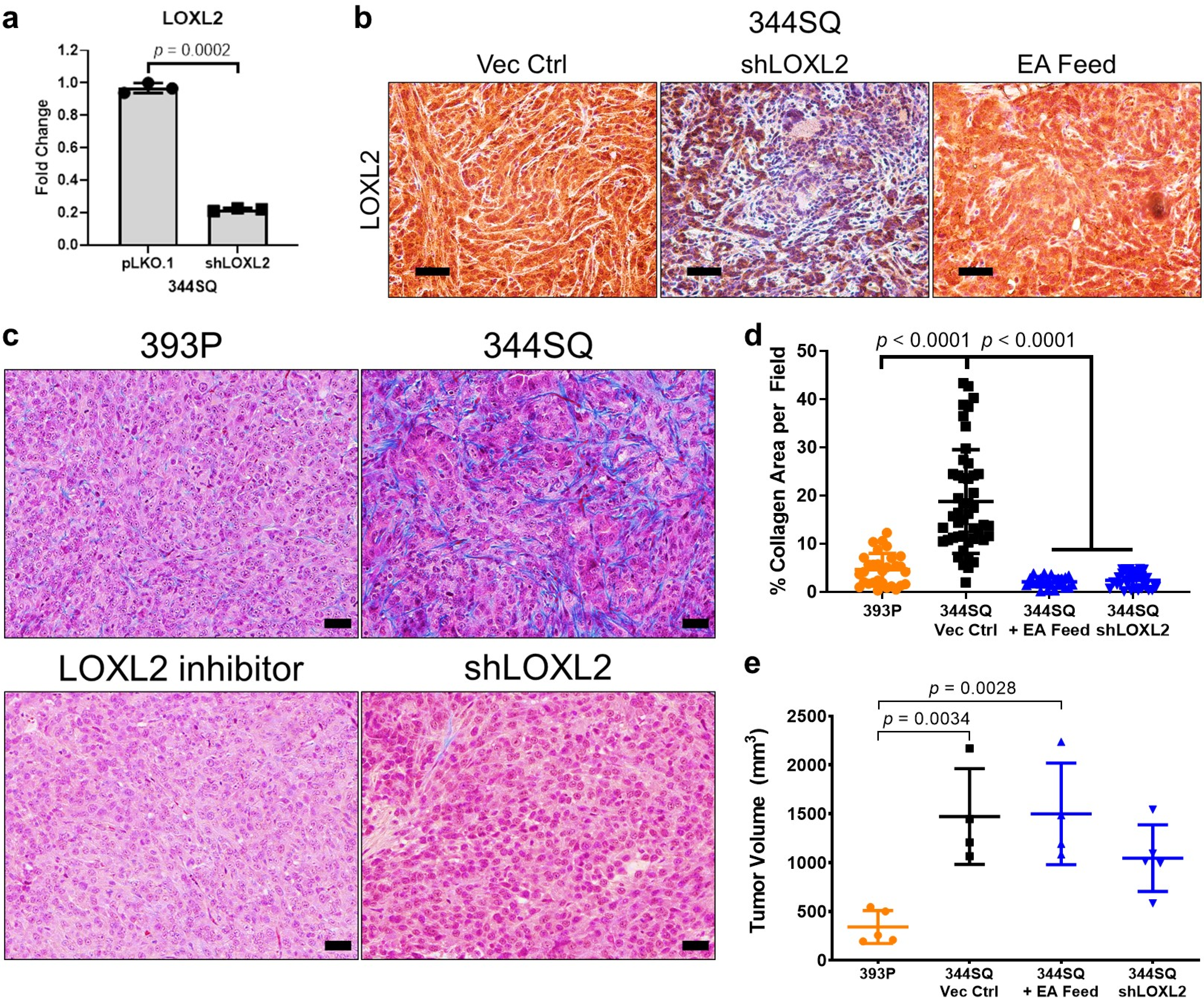

Supplementary Figure 2. LOXL2 knockdown or inhibition reduces intratumoral collagen crosslinking and deposition with no effect on tumor size

(a) QPCR of LOXL2 mRNA levels in 344SQ cells transduced with stable LOXL2 shRNA or vector control; n = 3 replicates per group. Data presented as mean +/- SD. Statistics calculated using two-tailed student's t-test. Representative qPCR data repeated over three independent runs.

(b) Representative LOXL2 IHC images of 344SQ tumors with stable LOXL2 shRNA knockdown or treated with ellagic acid (EA) feed from the experiment in Fig. 2. Scale bar, 50 µm.

(c – d) Representative trichrome stain images **(c)** and quantification **(d)** of 393P or 344SQ KP tumors with LOXL2 inhibition or knockdown; 393P and 344SQ-shLOXL2 n = 5 tumors with 6 fields quantified per tumor (30 fields total); 344SQ Vec Ctrl n = 4 tumors with 47 total fields quantified; 344SQ EA Feed n = 4 tumors with 26 total fields quantified. Scale bars, 50 µm. Data presented as mean +/- SD. Statistics calculated using one-way ANOVA post-hoc Tukey test.

(e) Quantification of final primary tumor volumes for indicated tumor groups from the experiment in Fig. 2a; 393P and 344SQ-shLOXL2 n = 5 mice per group; 344SQ Vec Ctrl and EA feed n = 4 mice per group. Data presented as mean +/- SD. Statistics calculated using one-way ANOVA post-hoc Tukey test.

Supplementary Figure 3. Flow cytometry sample gating strategy for live T cell populations in 344SQ tumors

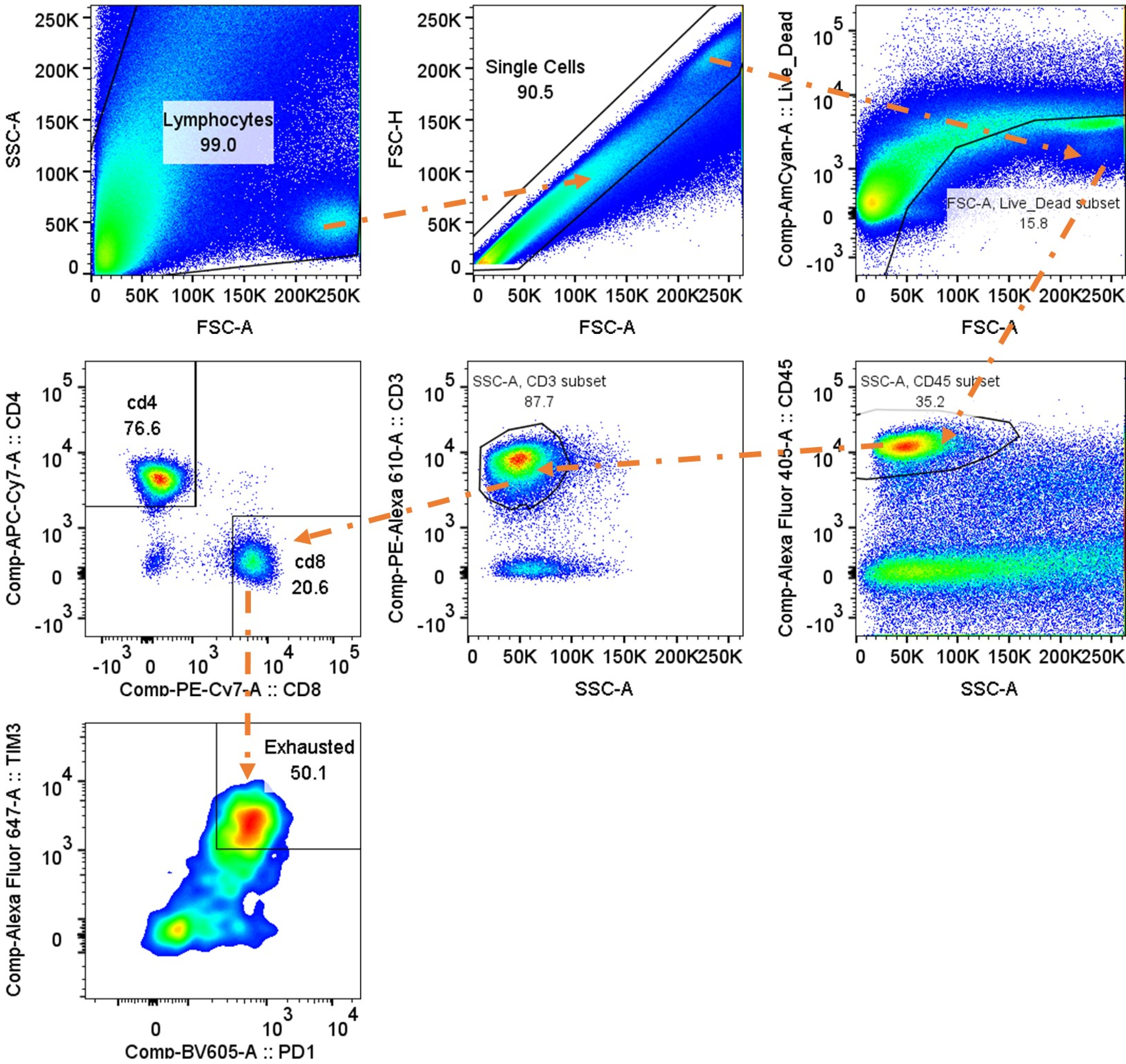

Supplementary Figure 3. Flow cytometry sample gating strategy for live T cell populations in 344SQ tumors

Tumor cell suspensions were gated for total cells (FSC-A vs. SSC-A) → cell singlets (FSC-A vs. FSC-H) → Live cells (FSC-A vs. Live/Dead) → $CD45^+$ cells (SSC-A vs. CD45) → $CD3^+$ cells (SSC-A vs. CD3) → $CD8^+CD4^-$ cells (CD8 vs. CD4) → PD-1+TIM-3+ cells (PD-1 vs. TIM3).

Supplementary Figure 4. Tumors with decreased collagen do not exhibit consistent or significant changes in other immune subpopulations

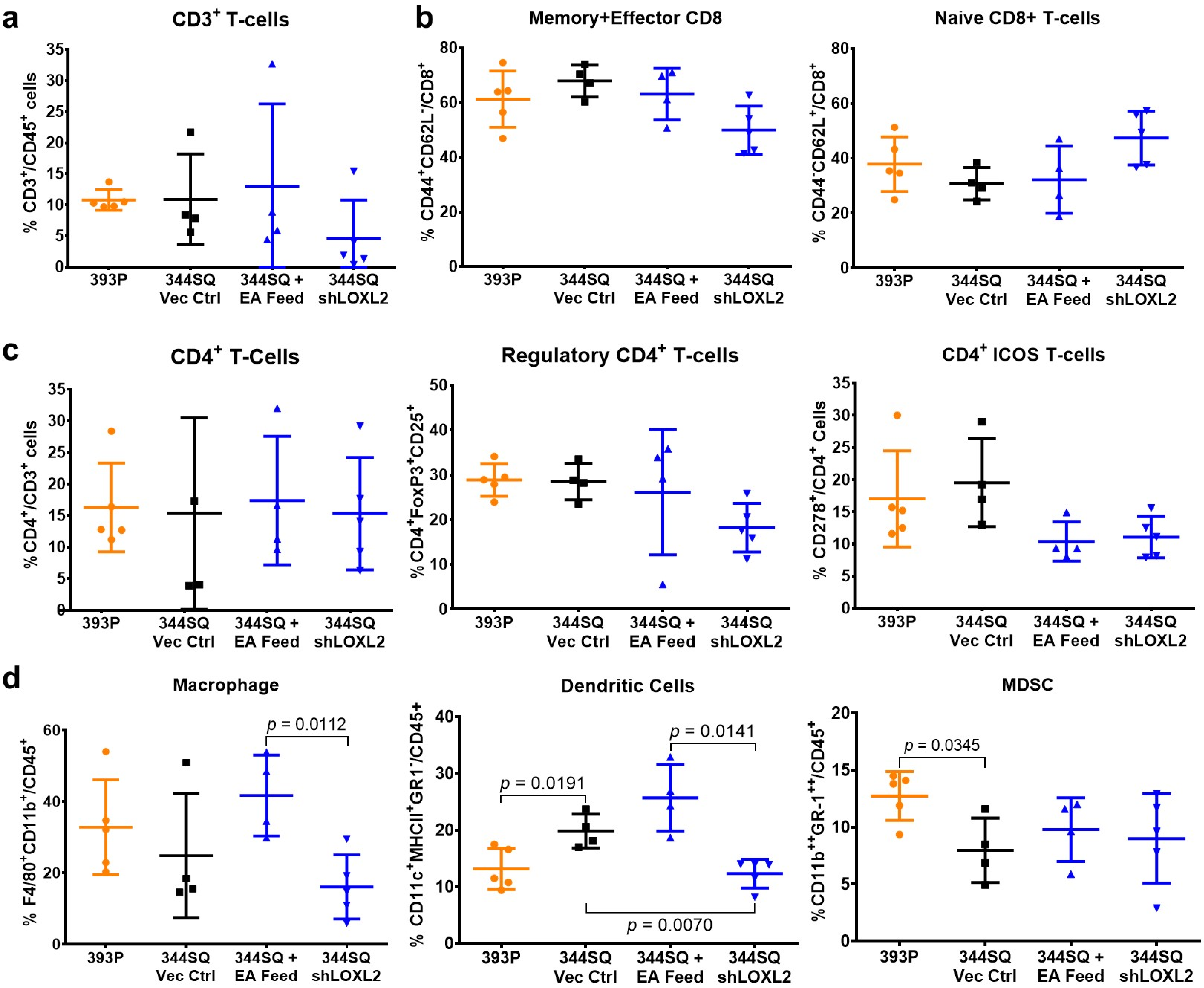

Supplementary Figure 4. Tumors with decreased collagen do not exhibit consistent or significant changes in other immune subpopulations

(a – d) Quantification of FACS percentages of specified tumor cell suspensions for **(a)** total CD3+ T cells; **(b)** memory/effector and naïve CD8+ T cells gated from total $CD45^{+}CD3^{+}CD8^{+}$ populations in Fig. 2c; **(c)** regulatory and ICOS $CD4^{+}$ T cells gated from total $CD45^{+}CD3^{+}CD4^{+}$ populations; and **(d)** indicated APC subpopulations; n = 5 biological replicates for 393P tumors and 4 biological replicates for each 344SQ tumor treatment group. All data presented as mean +/- SD. Statistics calculated using one-way ANOVA post-hoc Tukey test.

Supplementary Figure 5. LOXL2 knockdown reduces collagen deposition and sensitizes tumors to PD-L1 blockade

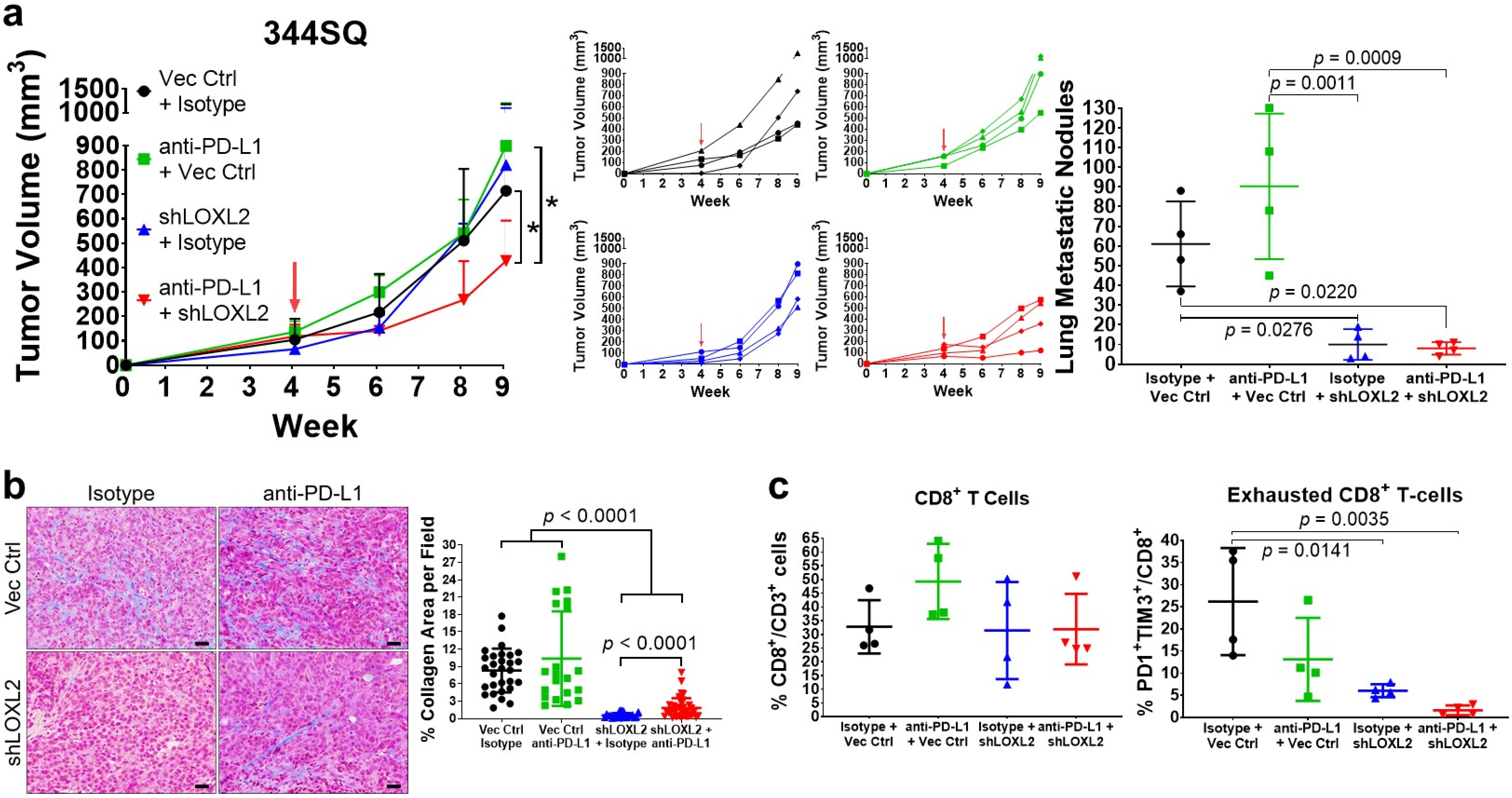

Supplementary Figure 5. LOXL2 knockdown reduces collagen deposition and sensitizes tumors to PD-L1 blockade.

(a) Left: Tumor volume measurements at indicated time points for 344SQ subcutaneous tumors with LOXL2 knockdown treated weekly with anti-PD-L1 (200 µg/mouse) or isotype control (200 µg/mouse). Starting time of PD-L1 blockade denoted by red arrow. Middle: Tumor volume measurements for individual mice in each treatment group from aforementioned experiment. Right: Quantification of lung metastatic surface nodules in indicated treatment groups at endpoint of experiment; n = 4 mice per treatment group.

(b) Representative trichrome stains including quantification of percent collagen area per field of 344SQ tumors in the indicated treatment groups at the endpoint of the experiment from **(a)**; n = 4 tumors per treatment group with 29 (Vec Ctrl + Isotype), 20 (Vec Ctrl + anti-PD-L1), 22 (shLOXL2 + Isotype), and 39 (shLOXL2 + anti-PD-L1) total fields analyzed across all tumors in respective groups. Scale bars, 50 µm.

(c) Quantification of FACS percentage of total $CD8^+$ (left) and $PD\text{-}1^+TIM\text{-}3^+$ exhausted (right) $CD8^+$ TILs gated from $CD3^+$ cell suspensions for individual tumor samples from the experiment in **(a)**; n = 4 tumors per group. All data presented as mean +/- SD and statistics calculated using one-way ANOVA post-hoc Tukey test. *$p < 0.05$.

Supplementary Figure 6. LOXL2 knockdown increases CD8 TILs in primary and metastatic lung tumors

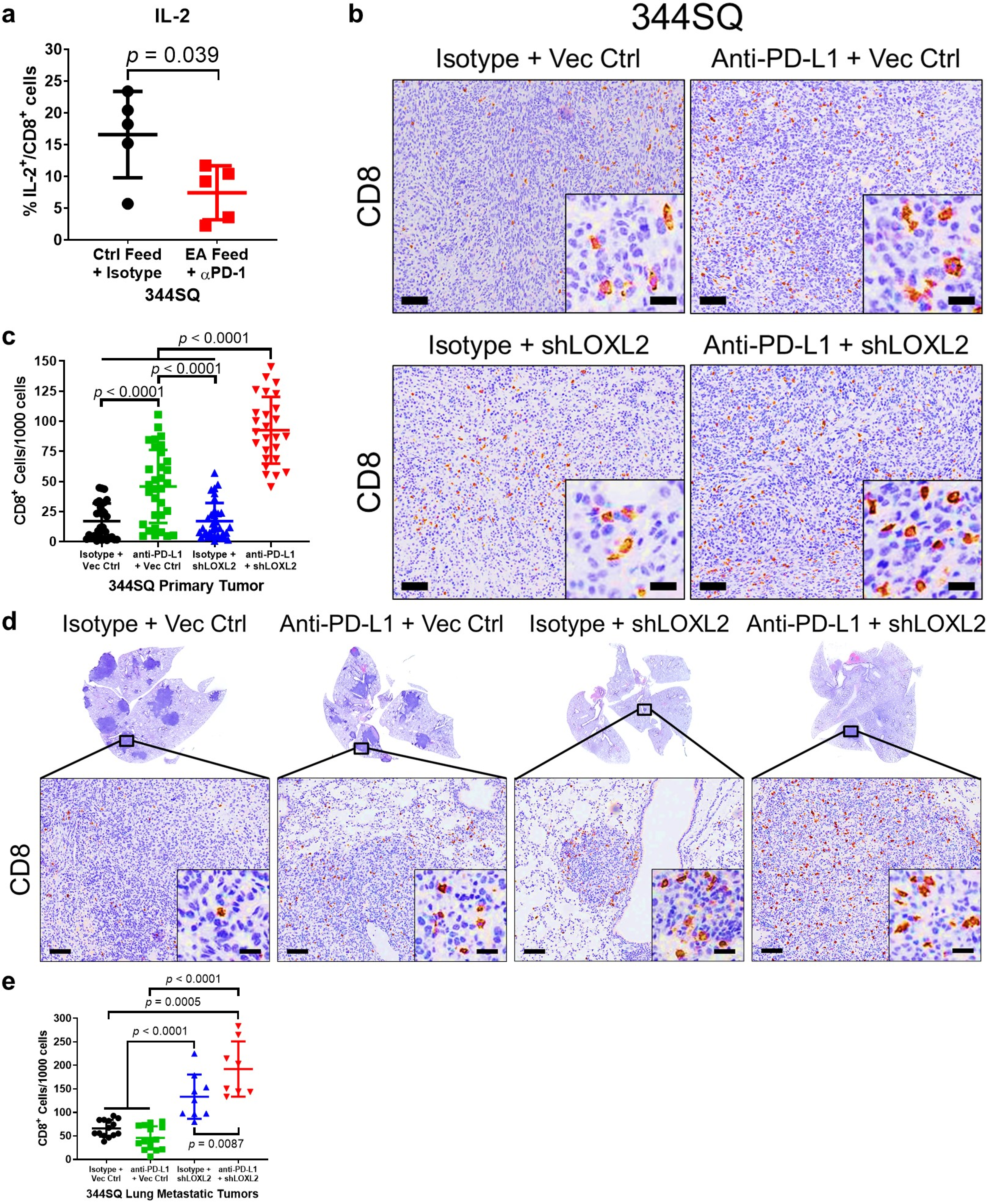

Supplementary Figure 6. LOXL2 knockdown increases CD8 TILs in primary and metastatic lung tumors

(a) FACS percentage of intracellular IL-2+ CD8+ TILs in tumor cell suspensions from experiment in Fig. 3d. Statistics calculated using two-tailed student's t-test.

(b – c) Representative CD8 IHC stains **(b)** with quantification **(c)** of 344SQ primary tumor tissues from the experiment in Supplementary Figure 5a; n = 4 tumors per group, 8 fields quantified per sample (32 total fields) for Isotype + Vec Ctrl, anti-PD-L1 + Vec Ctrl, and Isotype + shLOXL2 groups; 28 total fields quantified for anti-PD-L1 + shLOXL2 group. Scale bars, 100 µm. Inset scale bars, 20 µm. Statistics calculated using one-way ANOVA post-hoc Tukey test.

(d – e) Representative CD8 IHC stains **(d)** with quantification **(e)** of metastatic lung tumor regions as denoted by black zoom box from the experiment in Supplementary Figure 5a; n = 4 lungs per group with 14 (Isotype + Vec Ctrl), 15 (anti-PD-L1 + Vec Ctrl), 9 (Isotype + shLOXL2), and 8 (anti-PD-L1 + shLOXL2) lung tumor fields quantified across all samples for respective groups. Scale bars, 100 µm. Inset scale bars, 20 µm. All data plots presented as mean +/- SD. Statistics calculated using one-way ANOVA post-hoc Tukey test.

Supplementary Figure 7. LOXL2 inhibition sensitizes LLC-JSP tumors to PD-L1 blockade

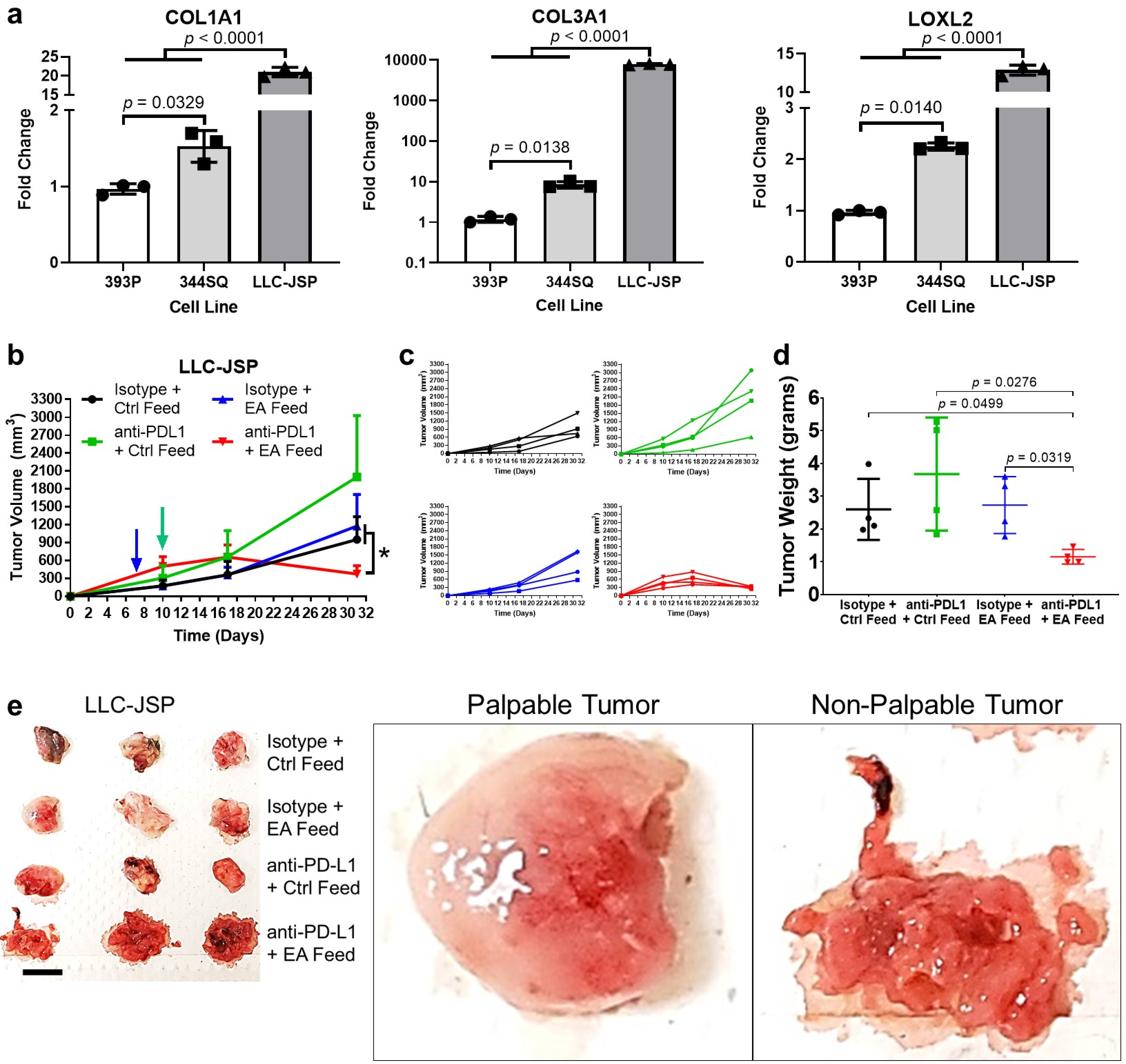

Supplementary Figure 7. LOXL2 inhibition sensitizes LLC-JSP tumors to PD-L1 blockade

(a) QPCR analysis for relative expression of COL1A1, COL3A1, and LOXL2 in 393P, 344SQ, and Lewis lung cancer LLC-JSP cell lines; n = 3 replicates per group.

(b) Time point tumor volume measurements for LLC-JSP subcutaneous tumors in indicated treatment groups. Tumors were injected in the right flanks of immunocompetent, syngeneic WT BL/6 mice. Mice were given EA feed 1 week following tumor implantation as denoted by the blue arrow. When tumors reached ~150 to 200 mm^3, anti-PD-L1 or isotype control was administered weekly (200 µg/mouse) by I.P. injection for 3 weeks as denoted by green arrow; n = 4 mice per treatment group.

(c) Tumor volume measurements for individual mice in each treatment group from the experiment in **(b)**.

(d) Final tumor weights of LLC-JSP tumors in indicated treatment groups from the experiment in **(b)**.

(e) Left: Images of three representative LLC-JSP primary subcutaneous tumors for each of the indicated treatment groups; n = 4 tumors per treatment group. Scale bar, 1 cm. Right: Enhanced representative images of palpable and non-palpable tumors from mice that received EA feed + isotype control or EA feed + anti-PD-L1, respectively. All data plots presented as mean +/- SD and statistics calculated using one-way ANOVA post-hoc Tukey test. *$p < 0.05$.

Supplementary Figure 8. Collagen reduces total CD8+ T cells and increases CD8+ T cell exhaustion and LAIR1 expression

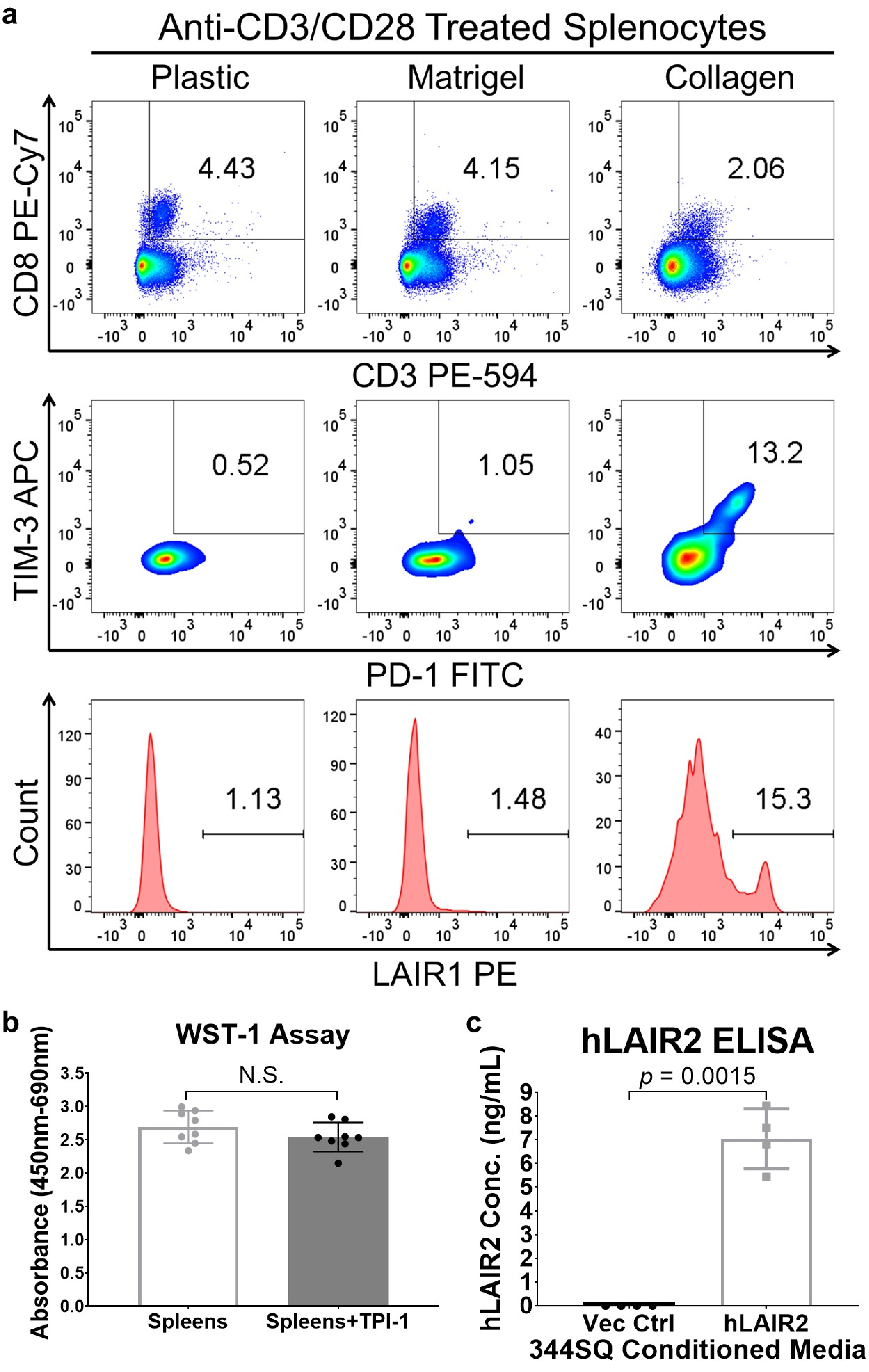

Supplementary Figure 8. Collagen reduces total CD8$^+$ T cells and increases CD8$^+$ T cell exhaustion and LAIR1 expression

(a) Representative FACS plots CD8$^+$ T cells gated from CD45$^+$CD3$^+$ splenocytes co-cultured on plastic, laminin-rich Matrigel, or collagen for 96 hours. CD8$^+$ T cells were then gated for PD-1$^+$TIM-3$^+$ exhaustion markers and LAIR1 expression and percentages were quantified in Fig. 4i – k.

(b) *In vitro* cell survival response of splenocytes after 72 hours of 20 µM SHP-1 (TPI-1) treatment. Cells were quantified using WST-1 reagent; n = 8 replicates per treatment group.

(c) LAIR2 ELISA for human LAIR2 (hLAIR2) concentration in conditioned media from 344SQ cells constitutively expressing LAIR2 or vector control; n = 4 independent samples. All data presented as mean +/- SD. Statistics calculated using two-tailed student's t-test.

Supplementary Figure 9. Lung cancer cell-induction of CD8+ T cell exhaustion and LAIR1 expression is dependent on LOXL2 and SHP-1 signaling due to CD18 binding to collagen

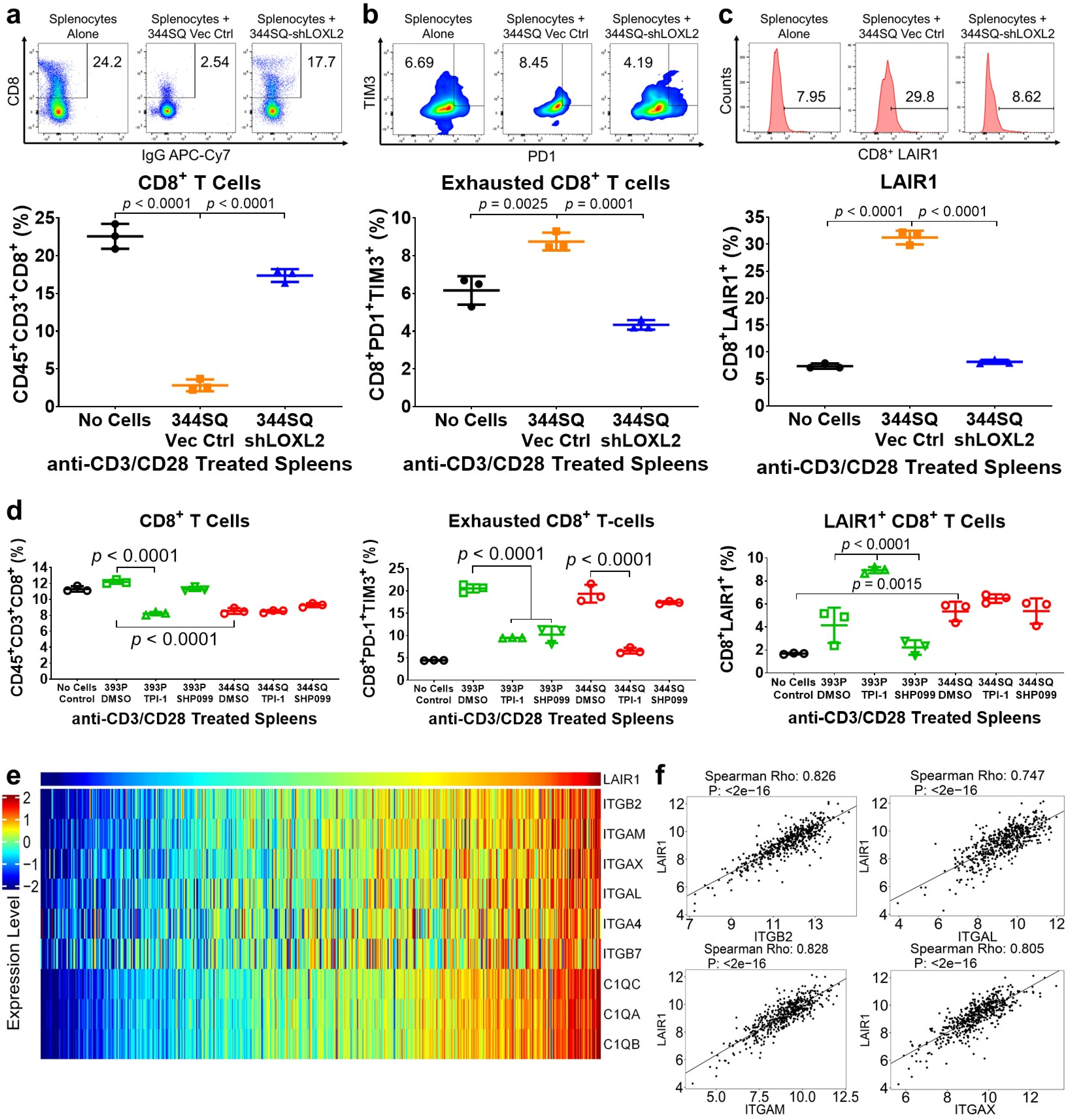

Supplementary Figure 9. Lung cancer cell-induction of $CD8^+$ T cell exhaustion and LAIR1 expression is dependent on LOXL2 and SHP-1 signaling due to CD18 binding to collagen

(a) Representative FACS plots and quantification of percentage of total $CD8^+$ T cells gated from $CD45^+CD3^+$ splenocytes in *in vitro* culture alone or in co-culture with 344SQ cells ± LOXL2 knockdown for 96 hours; n = 3 replicates per group. Data presented as mean +/- SD.

(b) Representative FACS plots and quantification of percentage of $PD\text{-}1^+TIM\text{-}3^+$ exhaustion markers gated from total $CD8^+$ T cells in splenocytes cultured alone or co-cultured with 344SQ cells ± LOXL2 knockdown for 96 hours; n = 3 replicates per group. Data presented as mean +/- SD.

(c) Representative FACS plots and quantification of percentage of $LAIR1^+$ cells gated from $CD8^+$ T cells in splenocytes cultured alone or co-cultured with 344SQ cells ± LOXL2 knockdown for 96 hours; n = 3 replicates per group. Data presented as mean +/- SD.

(d) FACS percentage of total $CD8^+$ T cells, $PD\text{-}1^+TIM\text{-}3^+$ exhausted $CD8^+$ T cells, and $LAIR1^+$ $CD8^+$ T cells in splenocytes cultured alone or co-cultured with 393P and 344SQ cells treated with DMSO, 10 μM TPI-1, or 20 μM SHP099 for 96 hours; n = 3 replicates per group. Data presented as mean +/- SD. Statistics calculated using one-way ANOVA post-hoc Tukey's test.

(e) Heatmap showing association of mRNA expression in TCGA LUAD dataset between LAIR1 and collagen receptor genes that are statistically significant ($P < 0.05$) by Spearman's rank correlation.

(f) Representative cluster plot analysis of Spearman's rank correlation between ITGB2, ITGAL, ITGAM, and ITGAX versus LAIR1 mRNA expression in TCGA LUAD dataset.

Supplementary Figure 10. Combination of PD-1 blockade with LAIR2 overexpression or SHP-1 inhibition reduces lung tumor growth and metastasis

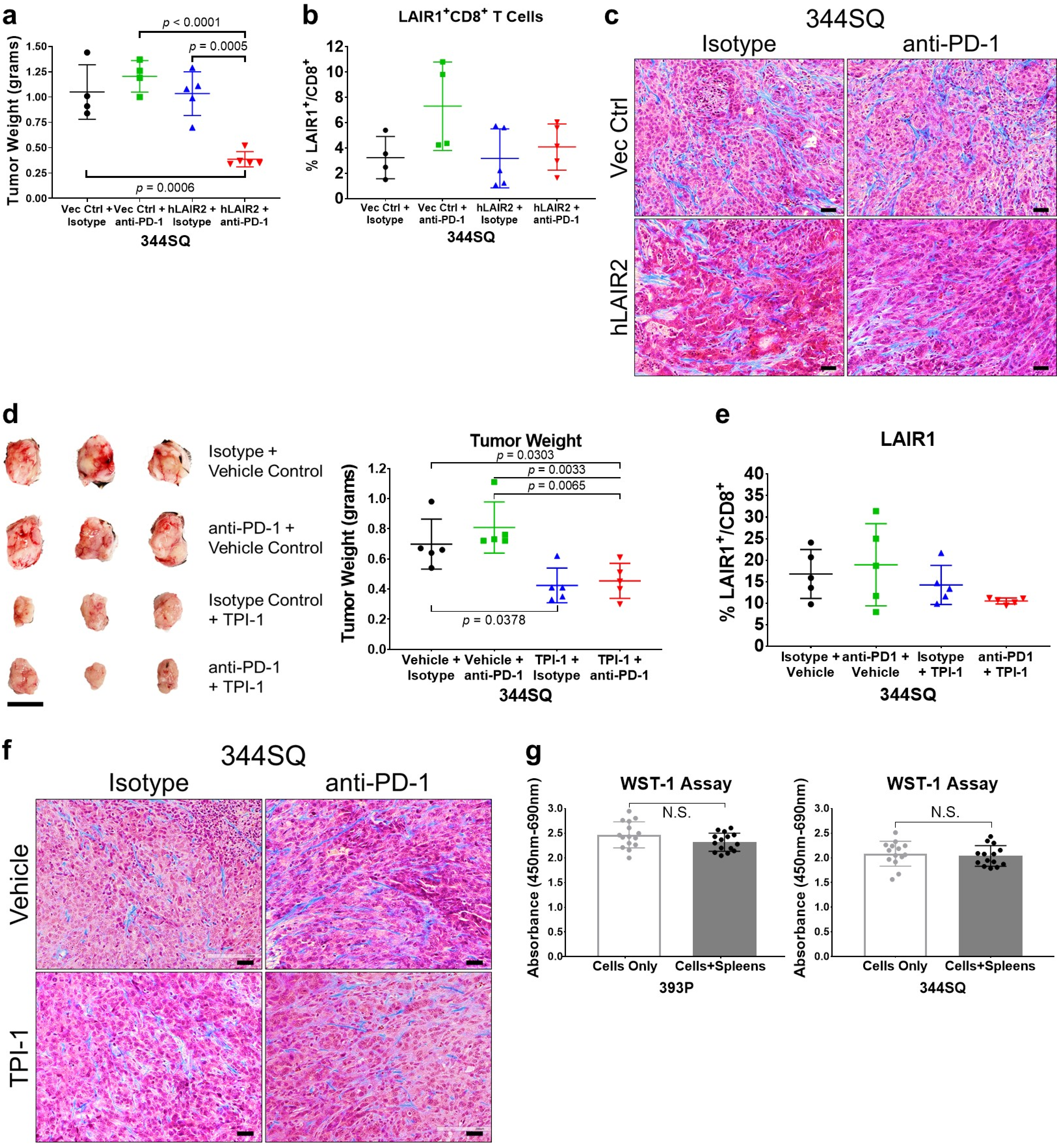

Supplementary Figure 10. Combination of PD-1 blockade with LAIR2 overexpression or SHP-1 inhibition reduces lung tumor growth and metastasis

(a) Final tumor weights of 344SQ tumors in specified treatment groups from the experiment in Fig. 5a; n = 4 or 5 tumors in each treatment group as denoted by individual dots. Statistics calculated using one-way ANOVA post-hoc Tukey test.

(b) FACS quantification for percentage of $LAIR1^+$ expression gated from total $CD8^+$ T cells in specified 344SQ tumor cell suspensions from the experiment in Fig. 5a; n = 4 or 5 tumors in each treatment group as denoted by individual dots.

(c) Representative trichrome stains of 344SQ tumors with indicated treatment groups at the endpoint of the experiment from Fig. 5a; n = 5 tumors per treatment group. Scale bars, 50 µm.

(d) Left: Images of three representative 344SQ primary subcutaneous tumors for each of the indicated treatment groups from the experiment in Fig. 6a. Right: Final tumor weights of 344SQ tumors in specified treatment groups from the experiment in Fig. 6a; n = 5 tumors in each treatment group. Statistics calculated using one-way ANOVA post-hoc Tukey test.

(e) FACS quantification for percentage of $LAIR1^+$ expression gated from total $CD8^+$ T cells in specified 344SQ tumor cell suspensions from the experiment in Fig. 6a; n = 5 tumors in each treatment group.

(f) Representative trichrome stains of 344SQ tumors in the indicated treatment groups at the endpoint of the experiment from Fig. 6a; n = 5 tumors per treatment group. Scale bars, 50 µm.

(g) WST-1 signal from 393P and 344SQ cells in culture alone or in co-culture with splenocytes without drug treatment from experiment in Fig. 6f. All data presented as mean +/- SD. Statistics calculated using two-tailed student's t-test.

Supplementary Figure 11. Human cancer patient mRNA expression dataset correlations and comparisons

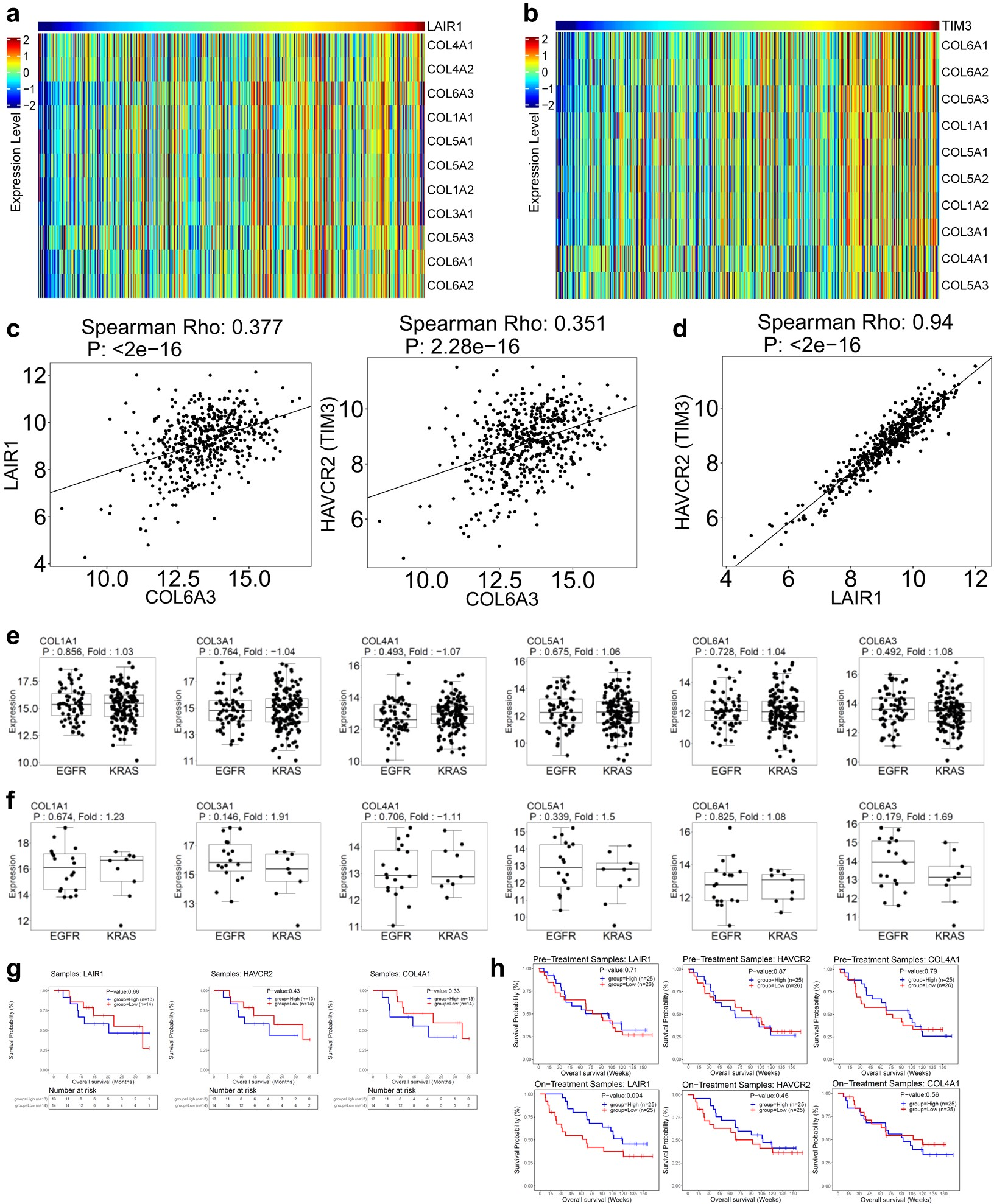

Supplementary Figure 11. TCGA lung cancer mRNA expression dataset correlations and comparisons

(a) Heatmap showing association of mRNA expression in TCGA LUAD dataset between LAIR1 and collagen genes that are statistically significant ($P < 0.05$) by Spearman's rank correlation.

(b) Heatmap showing association of mRNA expression in TCGA LUAD dataset between HAVCR2 (TIM-3) and collagen genes that are statistically significant ($P < 0.05$) by Spearman's rank correlation.

(c) Representative cluster plot analysis of Spearman's rank correlation between COL6A3 versus LAIR1 or HAVCR2 (TIM-3) mRNA expression in TCGA LUAD dataset.

(d) Cluster plot analysis of Spearman's rank correlation between LAIR1 versus HAVCR2 (TIM-3) mRNA expression in TCGA LUAD dataset.

(e – f) Comparison of mRNA expression of indicated collagen isoforms between mutant EGFR vs KRAS LUAD **(e)** and LUSC **(f)** TCGA datasets. LUAD mutant EGFR sample size n = 114 and mutant KRAS sample size n = 158 independent patient samples. LUSC mutant EGFR sample size n = 18 and mutant KRAS sample size n = 9 independent patient samples. Boxplots shown as the median ± 1 quartile, with whiskers extending to the most extreme data point within 1.5 interquartile range from the box boundaries.

(g) Kaplan-Meier curves predicting survival of melanoma patients receiving anti-PD-1 therapy from the dataset published by Hugo et al based on net mRNA level changes in LAIR1, HAVCR2 (TIM3), and a representative collagen gene (COL4A1) in pre-treatment biopsy samples.

(h) Kaplan-Meier curves predicting survival of melanoma patients receiving anti-PD-1 therapy from the dataset published by Riaz et al based on net mRNA level changes in LAIR1, HAVCR2 (TIM3), and a representative collagen gene (COL4A1) in pre- and on-treatment biopsy samples.

Supplementary Methods

Mouse LOXL2 shRNA Primers:

Primer	Sequence
mLOXL2-shRNA Forward	5’-TCGAGGCAACATGCTCTCTATCGGTTGTTGCGGCCGCAGACAACCGATAGAGAGCATGTTGCCTTTTTG-3’
mLoxL2-shRNA Reverse	5’-CGCGCAAAAAGGCAACATGCTCTCTATCGGTTGTCTGCGGCCGCAACAACCGATAGAGAGCATGTTGCC-3’

Human LAIR2 cDNA Primers for ectopic experiments:

Primer	Sequence
hLAIR2-BamHI-F	5’- gggaccggatccATGTCTCCACACCTCACTGCTCT-3’
hLAIR2-XhoI-R	5’- ATTTCTctcgagTCATGGTGCATCAAATCCGGAGGCTT-3’

QPCR Primers:

Primer	5’-Sequence-3’
mLOXL2-5’	TTCTGCCTGGAGGACACTGAGT
mLOXL2-3’	TCGGTGATGTCTATCCACTGGC
mCol1a1-F-1	CCTCAGGGTATTGCTGGACAAC
mCol1a1-R-1	CAGAAGGACCTTGTTTGCCAGG
mCol3a1-F-1	GACCAAAAGGTGATGCTGGACAG
mCol3a1-R-1	CAAGACCTCGTGCTCCAGTTAG
mL32-F	GGAGAAGGTTCAAGGGCCAG
mL32-R	TGCTCCCATAACCGATGTGT

Antibodies:

Antigen	Vendor	Catalog Number	Application	Dilution
Mouse/Human LOXL2	R&D Systems	AF2639	WB	1:250
Collagen I	Abcam	ab34710	WB	1:500
β-Actin	Sigma-Aldrich	A1978	WB	1:5000
Mouse LOXL2	Santa Cruz	sc-66950 (H-65)	IHC	1:200
CD8	Cell Signaling	98941	IHC	1:100
CD45 Pacific Blue	Biolegend	103126	FACS	1:100
CD3 PE-594	Biolegend	100246	FACS	1:100
CD4 APC-Cy7	Biolegend	100526	FACS	1:100
CD8 PE-Cy7	Biolegend	100721	FACS	1:200
CD44 BV711	Biolegend	103057	FACS	1:100
CD62L FITC	Tonbo Biosciences	35-0621-U500	FACS	1:100
CD69 BV650	Biolegend	104541	FACS	1:100
PD-1 BV605	Biolegend	135220	FACS	1:100
TIM-3 APC	Biolegend	134007	FACS	1:100
LAIR1 PE	Invitrogen	12-3051-82	FACS	1:100
Live/Dead Ghost Violet 510	Tonbo Biosciences	13-0870-T100	FACS	1:500
ICOS (CD278) BV786	Biolegend	313510	FACS	1:100
CD25 BV395	Biolegend	564022	FACS	1:100
FOXP3 PerCP-Cy5.5	Invitrogen	45-5773-82	FACS	1:100
CD11b BV650	Biolegend	101239	FACS	1:100

CD11c BV785	Biolegend	117335	FACS	1:100
GR-1 BV711	Biolegend	108443	FACS	1:100
F4/80 APC	Tonbo Biosciences	20-4801-U100	FACS	1:100
MHCII PE-Cy7	Biolegend	107629	FACS	1:100
CD45 PerCP-Cy5.5	Biolegend	103132	FACS	1:100
PD-1 FITC	Biolegend	135214	FACS	1:100
IL-2 BV605	Biolegend	503829	FACS	1:100
IFN-γ PE	Biolegend	505808	FACS	1:100

IHC Antibodies for Human Tissues:

Biomarker	Clone	Vendor	Catalogue #	Antigen Retrieval	Dilution
Collagen type I (COL1A1)	E8I9Z	Cell Signaling	91144	Epitope Retrieval #1 (Citrate Buffer ph6)	1:400
Collagen type III	polyclonal	Abcam	ab7778	Epitope Retrieval #1 (Citrate Buffer ph6)	1:400
TIM3	D5D5R	Cell Signaling	45208	Epitope Retrieval #2 (Tris-EDTA Buffer)	1:100
CD8	C8/144B	Thermo Scientific	MS-457s	Epitope Retrieval #1 (Citrate Buffer ph6)	1:25
