# Supplementary material for: Collagen promotes anti-PD-1/PD-L1 resistance in cancer through LAIR1-dependent CD8+ T cell exhaustion
Source: Nat Commun. 2020 Sep 9;11:4520. doi: 10.1038/s41467-020-18298-8 (PMC7481212; doi:10.1038/s41467-020-18298-8)
Supplement: Supplementary file 3 — Reporting summary [file 41467_2020_18298_MOESM3_ESM.pdf]

nature research

Corresponding author(s): Don L Gibbons

Last updated by author(s): Jul 26, 2020

Reporting Summary

Nature Research wishes to improve the reproducibility of the work that we publish. This form provides structure for consistency and transparency in reporting. For further information on Nature Research policies, seeAuthors & Referees and theEditorial Policy Checklist .

Statistics

For all statistical analyses, confirm that the following items are present in the figure legend, table legend, main text, or Methods section.

n/a	Confirmed	
☐	☒	The exact sample size (*n*) for each experimental group/condition, given as a discrete number and unit of measurement
☐	☒	A statement on whether measurements were taken from distinct samples or whether the same sample was measured repeatedly
☐	☒	The statistical test(s) used AND whether they are one- or two-sided<br>*Only common tests should be described solely by name; describe more complex techniques in the Methods section.*
☒	☐	A description of all covariates tested
☐	☒	A description of any assumptions or corrections, such as tests of normality and adjustment for multiple comparisons
☐	☒	A full description of the statistical parameters including central tendency (e.g. means) or other basic estimates (e.g. regression coefficient) AND variation (e.g. standard deviation) or associated estimates of uncertainty (e.g. confidence intervals)
☐	☒	For null hypothesis testing, the test statistic (e.g. *F*, *t*, *r*) with confidence intervals, effect sizes, degrees of freedom and *P* value noted<br>*Give P values as exact values whenever suitable.*
☒	☐	For Bayesian analysis, information on the choice of priors and Markov chain Monte Carlo settings
☒	☐	For hierarchical and complex designs, identification of the appropriate level for tests and full reporting of outcomes
☐	☒	Estimates of effect sizes (e.g. Cohen's *d*, Pearson's *r*), indicating how they were calculated

Our web collection on statistics for biologists contains articles on many of the points above.

Software and code

Policy information about availability of computer code

Data collection	GraphPad 7 was used to input data and generate graphs, FACSDiva 8.0 was used to collect FACS data, Aperio AT2 Scanner and ImageScope was used to scan and capture histology images, BioTek Epoch Microplate Spectrophotometer was used to measure colorimetric cell growth assays, R 3.5.1 was used to analyze RPPA, RNA profiling, and TCGA data. Westerns were developed by radiographic film. QPCR data were collected using Applied Biosystems 7500 Fast. Zeiss Zen System LSM 800 was used to capture second harmonics images. Analysis of IHC and trichrome mouse tissue stains were performed on ImageJ v1.53
Data analysis	GraphPad 7 was used to analyze fold changes and statistical data on graphs. R 3.5.1 was used to analyze RPPA, RNA profiling, and TCGA datasets. ImageJ v1.53 was used to quantify IHC, Masson's trichrome and SHG signal. FlowJo 10.6.1 was used to analyze FACS data. IHC of human patient samples were quantified using HALO (Indica Labs) Software.

For manuscripts utilizing custom algorithms or software that are central to the research but not yet described in published literature, software must be made available to editors/reviewers. We strongly encourage code deposition in a community repository (e.g. GitHub). See the Nature Research guidelines for submitting code & software for further information.

Data

Policy information about availability of data

All manuscripts must include a data availability statement. This statement should provide the following information, where applicable:

- Accession codes, unique identifiers, or web links for publicly available datasets
- A list of figures that have associated raw data
- A description of any restrictions on data availability

author.Human lung cancer patient data are available from the TCGA repository from https://portal.gdc.cancer.gov/. Treated human melanoma patient data are available in the GEO repository (GSE78220 and GSE91061) from https://www.ncbi.nlm.nih.gov/geo/. The source data for all relevant figures and supplementary figures are provided as a Source Data file with this paper. All other The data that support the findings of this study are available upon request to the corresponding author. A reporting summary for this article is available as a Supplementary Information file.

Field-specific reporting

Please select the one below that is the best fit for your research. If you are not sure, read the appropriate sections before making your selection.

- [x] Life sciences
- [ ] Behavioural & social sciences
- [ ] Ecological, evolutionary & environmental sciences

For a reference copy of the document with all sections, see nature.com/documents/nr-reporting-summary-flat.pdf

Life sciences study design

All studies must disclose on these points even when the disclosure is negative.

Sample size

For animal studies, to test the hypothesis that effective combinatorial treatment would reduce primary tumor growth by at least 30% with a variance of 10%, the minimal sample size for 90% power with statistical significance accepted at $\alpha = 0.05$ was four mice per experimental group. In vitro studies sample sizes were similarly determined from power analyses with similar models where N = 3 to 8 technical replicates. Human tissue microarray and TCGA analysis sample size was determined by analyzing as many samples available to use.

Data exclusions

Animals that did not develop tumors after implantation or induction or that died for reasons not related to tumor burden were not included in data analyses.

Replication

Mice treatment studies were performed once with indicated biological replicates based on minimum sample size obtained from power analyses to optimize cost of mice, animal housing, and reagents. In vitro experiments were performed with a minimum of three technical triplicates and repeated with two or more independent experimental replicates.

Randomization

When tumors reached appropriate size as reported in the Methods Section, mice were randomized into indicated treatment groups based on the cages in which mice were housed. For KP GEMM treatments, mice genders were also randomized so that near equivalent male and female mice were in control or treatment groups. In vitro experiments were not randomized. Covariates were not relevant as cells or samples were under identical controlled environments, cultured in the same dish at the same time, or were from isogenic lines with no other co-variates present.

Blinding

Mice treatments were not blinded since most of the experiments required daily treatments and treatment groups and mice cage numbers had to be known for investigators and only the primary or secondary author were the main investigators performing the treatments. In vitro experiments were not blinded since the primary author had the best technical expertise and knowledge of reagents necessary to perform the experiments.

Reporting for specific materials, systems and methods

We require information from authors about some types of materials, experimental systems and methods used in many studies. Here, indicate whether each material, system or method listed is relevant to your study. If you are not sure if a list item applies to your research, read the appropriate section before selecting a response.

Materials & experimental systems

n/a	Involved in the study	
[ ]	[x]	Antibodies
[ ]	[x]	Eukaryotic cell lines
[x]	[ ]	Palaeontology
[ ]	[x]	Animals and other organisms
[ ]	[x]	Human research participants
[ ]	[x]	Clinical data

Methods

n/a	Involved in the study	
[x]	[ ]	ChIP-seq
[ ]	[x]	Flow cytometry
[x]	[ ]	MRI-based neuroimaging

Antibodies

Antibodies used

Mouse/Human LOXL2 R&D Systems AF2639 WB 1:250

Collagen I Abcam ab34710 WB 1:500

β-Actin Sigma-Aldrich A1978 WB 1:5000

Mouse LOXL2 Santa Cruz sc-66950 (H-65) IHC 1:200

CD8
Cell Signaling
98941
IHC

1:100

CD45 Pacific Blue
Biolegend
103126
FACS
1:100

CD3 PE-594
Biolegend
100246
FACS
1:100

CD4 APC-Cy7
Biolegend
100526
FACS
1:100

CD8 PE-Cy7
Biolegend
100721
FACS
1:200

CD44 BV711
Biolegend
103057
FACS
1:100

CD62L FITC
Tonbo Biosciences
35-0621-U500
FACS
1:100

CD69 BV650
Biolegend
104541
FACS
1:100

PD-1 BV605
Biolegend
135220
FACS
1:100

TIM-3 APC
Biolegend
134007
FACS
1:100

LAIR1 PE
Invitrogen
12-3051-82
FACS
1:100

Live/Dead Ghost Violet 510
Tonbo Biosciences
13-0870-T100

FACS
1:500

ICOS (CD278) BV786
Biolegend
313510
FACS
1:100

CD25 BV395
Biolegend
564022
FACS
1:100

FOXP3 PerCP-Cy5.5
Invitrogen
45-5773-82
FACS
1:100

CD11b BV650
Biolegend
101239
FACS
1:100

CD11c BV785
Biolegend
117335
FACS
1:100

GR-1 BV711
Biolegend
108443
FACS
1:100

F4/80 APC
Tonbo Biosciences
20-4801-U100
FACS
1:100

MHCII PE-Cy7
Biolegend
107629
FACS
1:100

CD45 PerCP-Cy5.5
Biolegend
103132
FACS
1:100

PD-1 FITC
Biolegend
135214
FACS
1:100

IL-2 BV605
Biolegend

	503829<br>FACS<br>1:100<br><br>IFN-γ PE<br>Biolegend<br>505808<br>FACS<br>1:100
Validation	Antibodies have been validated either from genetic knockdown experiments described in the manuscript or validated by the manufacturer as stated on the website from the catalog numbers listed above or published references on the manufacturers' websites.

Eukaryotic cell lines

Policy information about cell lines

Cell line source(s)	393P and 344SQ murine KP cell lines were derived by our lab and extensively published in previous studies and described in more detail in the Methods section of the manuscript. Lewis Lung Cancer cell line was purchased commercially from ATCC rederived from previous publications described in the manuscript. HEK-293 cells were commercially obtained from ATCC.
Authentication	Cell lines were authenticated in previous publications when the cells were initially derived. No new authentications were performed in this study.
Mycoplasma contamination	All cell lines were tested to be mycoplasma negative and were tested monthly using commercial detection kits as described in the Methods section.
Commonly misidentified lines<br>(See ICLAC register)	No misidentified cell lines were used.

Animals and other organisms

Policy information about studies involving animals; ARRIVE guidelines recommended for reporting animal research

Laboratory animals	Mice were 129/sv and BL/6 strain. Mice were housed in ventilated cage enclosures in an environment maintained at 50% humidity with ambient temperatures range between 66oF to 78oF and 12-hour light/dark cycles. Male and female mice at 3 months of age were used for experiments.
Wild animals	Study did not involve wild animals
Field-collected samples	Study did not utilize field-collected samples.
Ethics oversight	The study protocol was evaluated and approved by the Institutional Animal Care and Use Committee (IACUC) at The University of Texas MD Anderson Cancer Center

Note that full information on the approval of the study protocol must also be provided in the manuscript.

Human research participants

Policy information about studies involving human research participants

Population characteristics	Lung cancer tissue samples and datasets were obtained from adult lung cancer patients encompassing all ages, genders, and races that could be obtained for analysis.
Recruitment	Patient tissue samples were recruited based on availability to obtain as many samples as possible.
Ethics oversight	MD Anderson Cancer Center Institutional Review Board

Note that full information on the approval of the study protocol must also be provided in the manuscript.

Clinical data

Policy information about clinical studies

All manuscripts should comply with the ICMJEguidelines for publication of clinical research and a completedCONSORT checklist must be included with all submissions.

Clinical trial registration	No clinical trial was performed in this study.

Study protocol	No clinical trial was performed in this study.
Data collection	No clinical trial was performed in this study.
Outcomes	No clinical trial was performed in this study.

Flow Cytometry

Plots

Confirm that:

- [x] The axis labels state the marker and fluorochrome used (e.g. CD4-FITC).
- [x] The axis scales are clearly visible. Include numbers along axes only for bottom left plot of group (a 'group' is an analysis of identical markers).
- [x] All plots are contour plots with outliers or pseudocolor plots.
- [x] A numerical value for number of cells or percentage (with statistics) is provided.

Methodology

Sample preparation	Cell samples were collected from primary murine subcutaneous tumor tissues, digested with collagenase/dispase solution, and passed through single-cell filters
Instrument	FACS data was collected using BD LSRFortessa flow cytometer.
Software	FACS data was collected using FACSDiva and analyzed using FlowJo.
Cell population abundance	FACS analysis was performed on each sample to a total cell number between 500,000 to 2,000,000 events with a threshold of 10,000 to increase quality of samples per event. Each gated population was sorted so that at least 500 cells for the furthest gated cell population was recorded to obtain satisfactory percentage of the cell population. FACS quality was also ensured using compensation controls and FMO controls to verify that observed and gated populations were accurate and distinct.
Gating strategy	All samples were gated for FSC/SSC, then gated for FSC-A/FSC-H, then gated for CD45+ cells. For T-cell populations, CD45+ were double-gated for CD3+ cells. CD45+CD3+ cells were then gated for CD4+ or CD8+ cells from the same populations. CD8+ cells were gated for the indicated populations listed in the graphs from the figures. For antigen presenting populations, CD45+ cells were gated for the populations indicated on the graphs in the figures. Gates were drawn from distinct, observable stained populations using prior FMO controls to indicate where to draw the positive gates that were separate from the negative populations.

- [x] Tick this box to confirm that a figure exemplifying the gating strategy is provided in the Supplementary Information.
